# Editorial Comment on “Therapeutic Options for Recurrence of Weight and Obesity-Related Complications After Metabolic and Bariatric Surgery: An IFSO Position Statement”

**DOI:** 10.1007/s11695-024-07494-w

**Published:** 2024-09-12

**Authors:** Marco Bueter, Ralph Peterli, Paulina Salminen

**Affiliations:** 1 University of Zurich, Zurich, Switzerland; 2 University Digestive Health Care Center, St Clara Hospital, University Hospital Basel, Clarunis, Basel, Switzerland; 3 University of Turku, Turku, Finland; 4 Turku University Hospital, Turku, Finland

**Keywords:** Revision surgery, Conversion surgery, Metabolic bariatric surgery, IFSO position statement

Obesity is not merely a risk factor, but a chronic disease requiring lifelong therapy. Thus, by definition, as any other chronic disease, obesity cannot be cured. Instead, the best achievable result by any therapy is to control the disease and to put it into complete or partial remission allowing patients an acceptable quality of life as long as possible. However, even if once controlled, in keeping with the chronic nature of obesity, long-term weight maintenance with ongoing effective treatment is required [1]. The used treatment may have a suboptimal clinical response or recurrent weight gain [2], but a relapse of initially controlled obesity-related complications, such as type 2 diabetes, can also occur.

If this happens, an escalation of therapy is indicated. Options for adjustments after primary metabolic bariatric surgery (MBS) include revisional surgery to enhance the existing procedure and/or conversional surgery to a more potent procedure as well as endoscopic or pharmacological options. Diet, lifestyle, and behavioral interventions are key components as part of the management, but alone they are not sufficient to lead to a substantial and durable control of severe obesity neither as primary nor secondary treatment, e.g., in cases of recurrent weight gain [1].

The newest International Federation for the Surgery on Obesity and Metabolic Disorders (IFSO) position statement summarizes and discusses the available therapeutic options for recurrent weight gain and relapse of obesity complications after MBS. To ensure comparability of clinical outcomes after different therapy approaches, the statement underlines the need to adhere to a standardized and uniform terminology and definitions. In addition, the statement provides an urgently needed overview over the available scientific evidence on the results of revisional and/or conversional surgery, endoscopy, and pharmacotherapy in cases of recurrent weight gain.

One main finding of the statement is that the current literature to support a wide use of revisional and/or conversional surgery as well as endoscopic measures in cases of suboptimal initial clinical response or recurrent weight gain after primary MBS is very limited and often characterized by methodological limitations. Further, without uniform standardized definitions, it is impossible to achieve high-quality data to create evidence-based treatment paradigms for the relapse of the chronic disease of obesity. Fortunately, first steps towards enabling higher-quality research have been recently taken both by the already completed international multidisciplinary expert consensus as well as the upcoming larger multi-specialty group tasked with developing a clinical definition of obesity, which is expected to be published in 2024 [3]. The active implementation of uniform definitions will pave the way for a multicenter randomized clinical trial to be designed to identify optimal treatment strategies for recurrent weight gain. Finally, the new obesity management medications (OMMs) with excellent weight-loss outcomes and acceptable safety profiles [4–6] will likely be important in treating patients with suboptimal clinical response and recurrent weight gain after MBS. However, the long-term results, safety profile, efficacy, and cost-benefit ratio for OMMs are yet to be demonstrated for both primary treatment and escalated therapy in the context of MBS.

## Data Availability

No datasets were generated or analysed during the current study.
